# Physicians’ Perspectives on COVID-19: An International Survey

**DOI:** 10.3390/healthcare8030250

**Published:** 2020-08-02

**Authors:** Alina Dima, Daniel Vasile Balaban, Ciprian Jurcut, Ioana Berza, Ruxandra Jurcut, Mariana Jinga

**Affiliations:** 1 Rheumatology Department, Colentina Clinical Hospital, 020125 Bucharest, Romania; alina_dima@outlook.com (A.D.); ioana_berza@yahoo.com (I.B.); 2 Internal Medicine, Gastroenterology and Cardiology Departments, Carol Davila University of Medicine and Pharmacy, 020021 Bucharest, Romania; rjurcut@gmail.com (R.J.); mariana_jinga@yahoo.com (M.J.); 3 Internal Medicine and Gastroenterology Department, Dr Carol Davila Central University Emergency Military Hospital, 010825 Bucharest, Romania; cjurcut@gmail.com; 4 Cardiology Department, Prof Dr CC Iliescu Institute of Cardiovascular Diseases, 022322 Bucharest, Romania

**Keywords:** COVID-19, physicians, questionnaire, doctors, SARS-CoV-2, survey

## Abstract

Coronavirus disease 2019 (COVID-19) has put a tremendous pressure over health care systems worldwide. Physicians were faced to fight this novel, emerging disease, without evidence-based recommendations. Our aim was to investigate physicians’ point of view regarding the new coronavirus disease. We designed an on-line survey with 30 questions to assess physicians’ perception of personal impact as well as epidemiology, clinical features, management, and outcome in COVID-19. A total of 194 physicians from 43 countries, of which 42.3% were male, 45.4% had more than 5 years’ experience and 10.8% were heads of department/professors, filled-out the questionnaire. Although 47.4% of the physicians were currently treating patients, over 80% thought that they might get in contact with COVID-19 patients. A total of 36.6% physicians thought that they are not and 30.9% were not sure if they were being protected by the ongoing procedures. A total of 21.1% of the doctors felt that they are avoided by the persons with whom they usually interact in daily life and 24.7% were isolated from the household members they usually live with. A total of 72.7% of physicians considered that COVID-19-free patients are currently neglected. The results of the current survey raise awareness about the impact of COVID-19 on physicians’ practice.

## 1. Introduction

The coronavirus disease 2019 (COVID-19) breakup started in December 2019 in Wuhan, China [1,2]. Genetic analysis shows that it is a beta-coronavirus linked to the severe acute respiratory syndrome (SARS) [1,2,3]. The new SARS coronavirus (SARS-CoV-2) was declared a pandemic by the World Health Organization (WHO) in March 2020 and became a public health emergency.

While most COVID-19 patients have only asymptomatic or mild disease, about 14% of COVID-19 patients develop severe acute respiratory pathology and need additional oxygen and a further 5% need admission to intensive care units [1].

In anticipation of effective antivirals and vaccines, one of the measures implemented to control the pandemic was social distancing. This affected people’s feelings and daily habits all over the world [4]. Physicians were also distressed by this change as well as by increased work burn-out during periods with an increased number of COVID-19 patients, exceeding the usual hospital capacity. Moreover, the medical community worldwide was confronted with the need to treat a new, unknown disease, overly aggressive in some patients in terms of both virulence and severity. COVID-19 patients’ management is challenging as current guidelines are lacking and some of the used therapies are only addressed to patients included in trials [1,3,5]. As well as this, medical information is rapidly and continuously being updated as new data are gathered while fighting this emerging disease.

In some units, due to the large number of COVID-19 patients hospitalized, and in others aiming at limiting the infection spread, measures like treating only urgent cases were taken. Protocols are changing repeatedly, and no consensus exists on the definition of urgent or life-threatening conditions or procedures [6]. Therefore, COVID-19-free patients’ access to medical assistance was limited, making it no longer possible to maintain the same standard of care [7,8].

The practicing physicians’ beliefs about and subjective reactions to the COVID-19 pandemic are especially important and could influence their quality of life as well as the quality of care. The aim of this study was to investigate physicians’ perspective on important features of COVID-19, including many topics where there are not yet validated data or definite answers (e.g., the pandemic’s personal impact on physicians, measures of protection, COVID-19 prevention, clinical and paraclinical picture, options of treatment, and prognosis).

## 2. Methods

### 2.1. Study Design, Survey Structure and Implementation

The research is a cross-sectional, questionnaire-based on-line research. After an English literature review, a questionnaire addressed to physicians regarding the main features of the COVID-19 pandemic was conceived by the study’s authors. The survey was administered only in English. The first part of the 30-questions survey assessed the demographic data (questions 1–6). The next section comprised questions related to epidemiologic factors related to COVID-19 (questions 7–12). Then, issues related to COVID-19 management were assessed, including disease prevention, diagnosis, and therapy (questions 13–20). The last part was intended to evaluate perspectives for future therapies as well as which sources are used for medical information (questions 21–30). The full questionnaire is available as Supplemental File S1.

The final version of the questionnaire was introduced on a dedicated platform that generated a link, used afterwards for distribution on the internet. The survey was shared by the authors via social networks (Facebook, Twitter, LinkedIn), distributed especially in large on-line groups of physicians; it opened on 24 April and data collection lasted for 7 days.

### 2.2. Study Participants

Eligible participants were physicians who voluntarily decided to take part in the survey and accessed it through social media. The first items of the questionnaire were designed as a filter with skip logic and used to select only physicians as respondents. Each respondent could field the questionnaire only once. The unfinished questionnaire reports were excluded from the final analysis.

### 2.3. Ethical Issues

The physicians willing to participate could open the link to the questionnaire page, where the introduction section presented the general structure of this research as well as the final goal of publishing the obtained results (see Supplemental File S1). The study participation did not pose any risk and was completely voluntary. Thus, filling out the survey also implied informed consent for inclusion in the study. Ethics approval was obtained from the Ethics Committee of the Carol Davila University of Medicine and Pharmacy, Bucharest, Romania (CH1/9484/2020).

### 2.4. Statistical Analysis

The statistical approach was descriptive and categorical variables were expressed as numbers and percentage (with 95% confidence intervals calculated by One-Sample Binomial Success and Clopper-Pearson test). The SPSS Statistics 25 software Armonk, NY, USA: IBM Corp was used.

## 3. Results

### 3.1. Demographic Data of the Study Participants

The answers of 194 physicians (42.3% male vs. 57.7% female) were analyzed in this study. The great majority (90.7%, 95% CI 85.7–94.4%) were physicians working in a clinical setting at this survey moment (see Table 1).

The survey included, in a well-balanced manner, respondents in different stages of professional training, namely 38 (19.6%) subjects were residents, interns or fellows, 47 (24.2%) were specialist physicians with less, and 88 (45.4%) with more, than 5 years’ experience, while 21 (10.8%) of the surveyed doctors were Professor or Head of Department.

The medical specialty of doctors who completed the questionnaire was as follows: 26 (13.4%) physicians specialized in Cardiology, 22 (11.3%) Rheumatology, 21 (10.8%) Family Medicine/General Practitioner, 19 (9.8%) Internal Medicine, 12 (6.2%) Pediatrics, 9 (4.6%) Psychiatry, 7 (3.6%) Pneumology, 6 (3.1%) Anesthesiology, 5 (2.6%) Gastroenterology, Intensive Care, Nephrology, Obstetrics and Gynecology, Radiology, 4 (2.1%) Emergency Medicine, 3 (1.5%) Dentistry, ENT, General Surgery, Infectious Diseases, or Neurology, 2 (1.0%) Cardiac Surgery, Neurosurgery, Ophthalmology, Orthopedics/ Trauma, Pathology, Vascular Surgery, Bariatric Surgery, or Integrative Medicine, and 1 (0.5%) Dermatology, Endocrinology, Epidemiology, Hematology, Laboratory Medicine, Thoracic surgery, Urology, Addiction, Clinical Pharmacology, Digestive and Endocrine, Occupational Health Service, or Podiatry (see Supplemental File S2).

With regard to the country where participants are currently practicing medicine, there were respondents from 43 countries from five continents, namely (in alphabetical order): 2 (1.0%) physicians from Albania, 1 (0.5%) Algeria, 2 (1.0%) Austria, 1 (0.5%) Bahrain, 1 (0.5%) Bangladesh, 7 (3.6%) Belgium, 1 (0.5%) Bolivia, 2 (1.0%) Bulgaria, 6 (3.1%) Canada, 1 (0.5%) Czech Republic, 1 (0.5%) Denmark, 29 (14.9%) France, 1 (0.5%) Georgia, 9 (4.6%) Germany, 1 (0.5%) Greece, 1 (0.5%) Guyana, 3 (1.5%) Hungary, 9 (4.6%) India, 12 (6.2%) Italy, 1 (0.5%) Jordan, 1 (0.5%) Liberia, 1 (0.5%) Lithuania, 1 (0.5%) Mexico, 8 (4.1%) Netherlands, 2 (1.0%) Norway, 1 (0.5%) Oman, 2 (1.0%) Pakistan, 3 (1.5%) Portugal, 3 (1.5%) Republic of Moldova, 22 (11.3%) Romania, 1 (0.5%) Saudi Arabia, 5 (2.6%) Serbia, 1 (0.5%) Somalia, 6 (3.1%) Spain, 4 (2.1%) Sweden, 3 (1.5%) Switzerland, 1 (0.5%) The former Yugoslav Republic of Macedonia, 3 (1.5%) Turkey, 2 (1.0) Ukraine, 10 (5.2%) United Kingdom of Great Britain and Northern Ireland, 22 (11.3%) United States of America, and 1 (0.5%) Zimbabwe (see Supplemental File S2).

### 3.2. Epidemiological and General Data

The percentage of doctors currently working in departments dedicated to or where patients with COVID-19 (47.4%, 95% CI 40.2–54.7%) are hospitalized was balanced by those not currently treating COVID-19. However, a much greater proportion of the physicians (82.0%, 95% CI 75.8–87.1%) thought that they might come into contact with SARS-CoV-2-infected patients (see Table 2).

A total of 41 respondents (21.1% 95% CI 15.6–27.6%) felt that they were avoided or rejected by the persons with whom they usually interact in daily life. Only 12 (6.3%, 95% CI 3.2–10.6%) of the survey’s participants already had COVID-19. However, there was also an important proportion of respondents (32.0%) who were not sure whether they had already contacted SARS-CoV-2.

Regarding the protective equipment, the hospital’s procedures in triage and differentiated pathways, only 36.6% (71 physicians) were confident that these were enough to protect. The others, approximately two-thirds, were either unsure (30.9%) or disbelieving that the secure measures were sufficient for protection (32.5%). Most of the doctors included, 141 respondents (72.7%), thought that COVID-19-free patients are currently medically neglected.

A total of 48 physicians (24.7%, 95% CI 19–31.7%) declared that they were currently isolated from the people with whom they usually live, while the rest, 74.2%, were not (see Table 2).

### 3.3. Clinical and Management Selected Options

More than four-fifths of the study respondents agreed that fever, cough, dyspnea, and anosmia/ageusia are symptoms that are attributable to COVID-19. A majority also named diarrhea (70.1%) and headache (64.4%) as part of the COVID-19 clinical picture (see Table 3).

The reverse-transcriptase (RT) polymerase chain reaction (PCR) SARS-CoV-2 of the nasopharyngeal secretion was selected by most of the doctors surveyed as a main diagnostic test, by 126 (64.9%) physicians. A total of 40 (20.6%) doctors also selected the chest-computed tomography (CT) as the diagnostic tool with the highest diagnostic accuracy.

More than 70% of the physicians interviewed considered that each one of the following associated comorbidities might have an important impact on the COVID-19 prognosis: chronic respiratory failure (89.2%), diabetes (85.6%), heart failure (82.0%), obesity (76.8%) and arterial hypertension (74.7%) (see Table 3).

Of the blood tests proposed as possible markers of outcome in COVID-19, the lymphocyte count was chosen by 64.4%, D-dimer by 64.4%, C-reactive protein (CRP) by 59.3%, and the SARS-CoV-2 viral load by 42.3% of the study subjects. Ten (5.2%) physicians noted other markers as determinants of COVID-19 outcome, namely interleukin-6 (IL-6), LDH, transaminase, creatine-phospho-kinase (CPK), and procalcitonin.

Virtually all study participants acknowledged the respiratory viral transmission, while 86.6% the contact with contaminated objects, as infectious pathways. About half of respondents also considered the fecal-oral route as a potential transmission route.

Most of the physicians do not think that there are any useful therapies for COVID-19 prevention—113 (58.2%) participants. The therapies that were considered prophylactic by around one fifth of the participants were as follows: Vitamin D (20.6%), Zinc (18%), Vitamin C (23.2%), Hydroxychloroquine (HCQ) (17.0%). Some other therapeutic principles were noted as having a preventive effect, like Azithromycin (AZA) (1%), Melatonin (1%), Colchicine (0.5%), Estradiol (0.5%), and Isoprinosine (0.5%).

Regarding an effective therapy for COVID-19, 48.5% selected the plasma from convalescent donors, 33% HCQ, 32% Remdesivir, while 28.4% selected AZA (see Table 3). Only 8.7% of doctors noted that other therapies that might be effective for COVID-19 treatment, namely Colchicine, Cyclosporine, Estradiol, Corticosteroids, granulocyte-macrophage colony-stimulating factor (GM-CSF) in aerosol, Favipiravir, Ivermectin, Zinc, Darunavir/Cobicistat. A total of 69 (35.6%) of the respondents considered that there is no currently available effective treatment for COVID-19. Considering the role of hemostasis in COVID-19, 56.7% of physicians considered that it plays a central part and should be targeted by anticoagulation (see Table 3).

A total of 13.4% of the respondents think that there is no pre-existing medication that might worsen the prognosis in COVID-19, while 61.3% identified the ongoing immunosuppressive treatment as a negative prognostic factor in COVID-19 patients (see Table 3).

### 3.4. Future Developments Regarding the COVID-19 Outcome

Regarding an effective vaccine, 23.2% of physicians thought that there will not be any vaccine useful for preventing COVID-19, while the rest are confident about an effective vaccine development, and most respondents (43.8%) think that this will last for at least 12 months (see Table 4).

Further, 26.3% of physicians surveyed did not believe that an effective treatment for COVID-19 will be developed, while 5.7% considered that a treatment will be identified in the next 3 months, 18.6% in the next 6–12 months, and 32.0% after at least 12 months.

Only 13.9% of the respondents believed that immunity post-COVID-19 is permanent and there will not be reinfections with SARS-CoV-2. As many as 30.9% of respondents think that there might be reinfections with the same viral strain in less than 6 months, while 36.6% after at least 12 months, and with a different, new, mutated viral strain (see Table 4).

Most of the surveyed doctors (64.4%) responded that it is not possible to eradicate COVID-19, while 26.3% considered that this is possible, but after an interval of at least 2 years.

Regarding a possible persistence of the SARS-CoV-2 in the human body, with subsequent periodic reactivation, 22.7% of physicians responded it is not possible, and 24.2% that is feasible, while half are not sure on this matter.

### 3.5. Source of Medical Information and General Data

A total of 54.6% of the doctors questioned considered that they were well informed regarding COVID-19, while, on the contrary, 20.6% believed that the amount of information is not enough. 24.2% were not sure whether the information they get is sufficient or not.

With regards to the sources available for medical and scientific information about COVID-19, the most accessed were medical journals (86.1%), scientific societies’ websites (82.5%), and internal hospital protocols in the workplace (65.5%) (see Table 5).

## 4. Discussion

### 4.1. Epidemiological and General Data

The survey presented here gathered physicians’ opinion regarding the COVID-19 pandemic and included 194 doctors from more than 30 countries or medical specialties. The distribution, both by gender or age and by professional degree, was well balanced, and the majority worked in a clinical setting at the time of the survey.

The SARS-CoV-2 incubation period might range between 1 and 14 days [9]. The infectious burden is higher in the symptomatic period, but transmission seems possible even in the preceding period. In one series, negative serial periods were reported in 12.6% of the 468 patients [10]. The human transmission is done via respiratory droplets, when another person is as near as 1 m or less [9]. The transmission might also occur through fomites from infected persons via contaminated surfaces [9]. Airborne transmission is presumed only in special conditions like aerosol generating procedures. In such situations, the droplet nuclei may persist in the air for long periods and may be transmitted from distances larger than 1 m [9]. SARS-CoV-2 was also identified in the feces of ill patients, which might imply the fecal route as a means of transmission [11], not proven yet [9]. A study on fecal shedding of SARS-CoV-2 RNA has proven the replication of the virus in enterocytes, which may account for the digestive COVID-19 symptoms, but this is followed by colonic inactivation of the virus, which makes stool specimens non-infective and the fecal-oral transmission route improbable [12]. However, this digestive presentation of COVID-19 may lead to a delayed diagnosis and has been reported to be associated with slower viral clearance [13]. The physicians’ knowledge reflected well the existing data, as 99% acknowledged respiratory transmission, 86.6% contact with contaminated surfaces and almost half the fecal-oral route. Patients with COVID-19 mild disease might present respiratory or non-specific symptoms like fever, cough, asthenia, myalgia, sore throat, dyspnea, nasal congestion, headache and, to a lesser extent, digestive involvement [1]. Anosmia and ageusia have also been reported as presenting or sole manifestation [14,15]. Severe pneumonia may worsen to acute respiratory distress syndrome (ARDS) [1]. The various presentations and complications of COVID-19 are reflected in the diverse specialties of the respondents, as physicians of all backgrounds were confronted with this condition. Even though 52.6% of doctors were not currently treating COVID-19 patients, 82% foresee professional contact with them. Interestingly, only a third of physicians feel protected by the available protective equipment and protocols.

### 4.2. Diagnosis

The RT-PCR assay is the gold-standard for COVID-19 diagnosis [16]. The SARS-CoV-2 RT-PCR showed positive specimens in 93% of bronchoalveolar lavage fluid, 46% of fibro bronchoscope brush biopsy, 72% sputum, 63% nasal swabs, 32% pharyngeal swabs, 29% feces, 1% blood, and 0% urine—specimens collected from 205 patients [11].

The SARS-CoV-2 RT-PCR from nasopharyngeal swabs might be positive even after 6 weeks, and it was questioned if this reflects persistence, testing errors, reinfections, or reactivations [17]. The SARS-CoV-2 RT-PCR false negative results [18] might be due to sample collection or transportation, or by errors in the laboratory methods used [16]. Therefore, a positive SARS-CoV-2 RT-PCR result weighs more than a negative one with regard to increased specificity [19]. The relative low sensitivity should not be forgotten, therefore a negative test cannot exclude the pathology, especially in patients with suggestive symptomatology [19]. Interestingly, only about two-thirds of respondents considered RT-PCR as the main diagnostic test.

The assessment of specific SARS-CoV-2 IgM and IgG by enzyme-linked immunoassay (ELISA) might be also assessed to avoid the false-negative cases and to highlight the immune response [10,17]. Only 4.1% of the survey’s respondents considered ELISA as a main diagnostic test.

Pulmonary involvement proved by chest CT as ground-glass opacities might play an auxiliary role in sustaining the COVID-19 diagnosis [20]. Even if the chest CT sensitivity for COVID-19 is good (97%), its specificity is low (56%) [20].

### 4.3. Impact of Comorbidities

Comorbidities identified as independent predictors for in-hospital death were as follows: chronic obstructive pulmonary disease (OR 2.96), coronary artery disease (OR 2.70), heart failure (OR 2.48), cardiac arrhythmia (OR 1.95) [21]. Diabetes and obesity were also identified as negative prognosis factors for progression and mortality [22,23]. On the contrary, the hepatic impairment, malignancies or renal pathology were not associated with COVID-19 prognosis [16]. Patients with respiratory chronic conditions are advised to continue their regular treatment and be closely watched as being at greater risk [24]. Physicians’ answers reflected quite accurately what is currently known, with a wide consensus of comorbidities impacting on prognosis.

### 4.4. Laboratory Markers

The inflammatory pathways are activated during COVID-19, and therefore parameters like C-reactive protein (CRP), ferritin, procalcitonin, fibrinogen, interleukin-6 (IL-6) were studied as prognostic factors [2]. Lymphocyte count has been proven to be correlated with severity and outcome [25]. The CRP and IL-6 are proposed as useful follow-up markers [26]. Further, an important increase in ferritin level in severe COVID-19 patients as well as systemic involvement draw attention to the similarities with the “hyperferritinemic syndrome”, that include macrophage activation syndrome (MAS), adult-onset Still’s disease (AOSD), catastrophic anti-phospholipid syndrome (CAPS) and septic shock [27]. Coagulation parameters were included in the COVID-19 management algorithms to allow the recognition of cases with a severe prognosis, and to define the conditions for hospital admission, as well as to establish anticoagulation principles [2,5]. Therefore, screening for D-dimer, prothrombin time, platelet count, and fibrinogen is recommended [5]. Above all, the D-dimer level was mainly evaluated in association with disease prognosis [28]. Two-thirds of the survey’s respondents confirmed the lymphocyte count and D-dimer as prognostic markers, followed by CRP and viral load. Moreover, more than half of the physicians considered that hemostasis plays a central role in the disease pathophysiology and should be targeted by anticoagulation, a notion that is increasingly stated in guidelines for hospitalized patients.

### 4.5. Management

At the moment of the questionnaire distribution, there was no specific treatment confirmed to be effective and generally recommended for COVID-19. The efforts in search for a treatment were probably higher than ever before, with the literature rapidly updating [3]. In patients with mild disease, hospitalization is not necessary, while in moderate or severe hospitalized patients, close monitoring with supportive interventions, including supplemental oxygen therapy, empiric antimicrobial treatment, mechanical ventilation, conservative fluid management, low molecular-weight heparin was considered from the beginning [1]. It is interesting to see what the opinion at the end of April 2020 was, when the studies in COVID-19 patients had just begun. Some national medical societies recommend therapies like HCQ with/out AZA, Lopinavir/Ritonavir, Tocilizumab, or convalescent plasma only in clinical trials, taking into account the knowledge gap [3]. There are two randomized clinical trials (RCT) that failed to prove HCQ benefits in COVID-19 [29,30]. Moreover, the association of HCQ with AZA was associated with adverse effects, especially in terms of QTc interval prolongation [31,32]. The Lopinavir/Ritonavir combination did not prove the beneficial effects over the standard supportive care [33]; it is associated with QTc prolongation too, but also with digestive adverse effects [3]. Other antiviral therapies like Remdesivir and Favipiravir were also proposed [34]. An interim analysis of the Adaptive COVID-19 Treatment Trial (ACTT), conducted in April 2020, indicated a faster recovery and a survival benefit of Remdesivir over placebo [35]. Severe COVID-19 can be accompanied by an important IL-6 increase, like previous emerged coronaviruses [26]. Tocilizumab was indicated with good results over COVID-19 prognosis [36]. As for other biologic therapies, one of the main adverse effects is the increased risk of serious infections. A recent systematic review argues in favor of convalescent plasma in COVID-19, with a good safety profile, symptomatology improvement and reduced mortality [37]. Later, lose-dose lung radiation in COVID-19 pneumonia was proposed considering the expected anti-inflammatory effects [38,39].

The diversity of these data, as well as the hope surrounding several therapeutic solutions, was well reflected by the physicians’ answers, which included plasma from convalescent donors, HCQ and Remdesivir as the top three efficient therapies for COVID-19 in the end of April 2020.

As the SARS-CoV-2 pulmonary invasion is mediated through the ACE2 receptor [15,21,40], theoretical concerns about the ongoing angiotensin converting enzyme (ACE) inhibitors and angiotensin II receptor blockers (ARB) over the COVID-19 prognosis were raised. However, in one study that included 8910 COVID-19 patients, the use of ACE inhibitors or ARBs was not associated with increased risk of in-hospital death [21]. The American Heart Association [41] and The European Society of Cardiology [42] advised that there are no clinical data to sustain either adverse outcomes or beneficial effects of the ACE inhibitors or ARBs drugs to date, therefore the anti-hypertensive treatment can continue safely. A quarter of our respondents chose ACE inhibitors as possibly worsening the prognosis of COVID-19 patients, reflecting the early inconsistencies in the published data.

The febrile syndrome is the innate response to infection meant to limit the viral replication, and antipyretic therapies might alter the outcome. Moreover, nonsteroidal anti-inflammatory drugs (NSAID) like Ibuprofen increase the ACE2 production and might theoretically facilitate the SARS-CoV-2 infection. The harms and benefits of NSAIDs in COVID-19 patients are still unclear, but Paracetamol is recommended as first line for the febrile syndrome in COVID-19 [43]. More than a third of the physicians named NSAIDs as possibly worsening prognosis.

### 4.6. Prevention

To our knowledge, the only available ways to prevent the SARS-CoV-2 infection are general measures, meaning isolation, covering the nose and mouth, washing hands, and cleaning touched surfaces. The precedent global pandemic, the Severe Acute Respiratory Syndrome (SARS) in 2002 and the Middle East Respiratory Syndrome (MERS) in 2011, caused also by a coronavirus with zoonotic origin, showed protective specific IgG for SARS-CoV lasting as long as 2 years [40]. For the MERS-CoV, the seroconversion was found in days 14–21 of the disease [40] and a delay in obtaining the antibodies’ response was related, in both SARS-CoV and MERS-CoV, with a poor prognosis [40].

At the beginning of April 2020, there were 115 vaccine proposals, of which 78 active projects, 72% developed by private industry [5]. There are five types of vaccine anti-COVID-19 that are currently in phase I clinical trials, including mRNA-1273 from Moderna, Ad5-nCoV from CanSino Biologicals, INO-4800 from Inovio and LV-SMENP-DC and pathogen-specific aAPC from Shenzhen Geno-Immune Medical Institute [44]. Great efforts are being made for its rapid development, manufacture scale, and global access, especially in low-resource regions [5,45]. The most difficult tasks remain the proof of clinical efficiency as well as production on large scale [45]. Most of the survey respondents believe that an effective vaccine will be available in more than 6–12 months.

The natural response to SARS-CoV-2 produces an increase in the T-helper cells with subsequent IgM and IgG production that bind SARS-CoV-2 [46]. The IgM secretion starts, probably within the first seven days of infection [47]. One reason that convalescent plasma might be efficient in severe COVID-19 cases is ability of the antibodies anti-SARS-CoV-2 to counterbalance and suppress viremia [48]. The immune response to SARS-CoV-2 seems, therefore, to be efficient and protective, but there are not enough data to determine how long the immune response might last. At the survey’s timepoint, as little as 13.9% of our respondents believed that post-COVID-19 immunity is permanent, while the others evoked mutated strains, or possibility of late reinfection.

Just over half of the surveyed doctors felt that they were well informed on the COVID-19 topic, with medical journals and scientific websites being the most accessed sources. However, 47.9% are also considering information from less reliable sources, like social media. The mass media and social networks are important in defining both the level of knowledge as well as general perception [4]. Awareness should be given to the fake news and misleading news that is widely accessed [4].

### 4.7. Limitations

The current research has some limitations. First, the number of physicians that fully filled-out the questionnaire was lower than expected and so we do not have a lot adjusted to a sample size. Overall, the response rate for on-line questionnaires that use internet platforms might be ten times lower when compared to face-to-face completed questionnaires [49,50], less than one in ten among those reached by the questionnaire link [50]. Not all areas of the world are well represented, which might be because in some areas, COVID-19 was not yet a serious threat in April 2020 (e.g., Latin America). However, we managed to include physicians from an important number of countries, different specialties and from all professional levels, therefore the results presented can represent a general opinion among physicians at the moment of the questionnaire release. It is also important to note that the information is changing quickly, so the results presented here capture the opinion of the end of April 2020, but it is important to remember the level of knowledge at that stage. Further, the responses are not from experts in the field; for a professional point of view, guidelines are developed, most of which are referred to here. Another limitation is that we did not directly perform an identity check-up, which is not allowed by GDPR regulations, but this results from the first questions responses.

## 5. Conclusions

To conclude, we present the results of an international survey reflecting physicians’ perception of several COVID-19 questions. Awareness should be given to the physicians’ concerns related to their safety. Even if efforts which are higher than ever before are made in the SARS-CoV-2 pandemic limitations, there is great concern among physicians as to whether they will succeed.

## Figures and Tables

**Table 1:** Demographic data of the study participants.

Questions 1–3 and 6 of the Physicians’ Perspectives on COVID-19 Questionnaire		
1	Are you a physician currently practicing medicine in a clinical setting?	
	Yes	176 (90.7%)
	No	18 (9.3%)
2	What is your age?	
	20–29 years	22 (11.3%)
	30–39 years	73 (37.6%)
	40–49 years	51 (26.3%)
	50–59 years	32 (16.5%)
	60–69 years	12 (6.2%)
	70–79 years	4 (2.1%)
3	What is your gender?	
	Male	82 (42.3%)
	Female	112 (57.7%)
6	What is your current professional level?	
	Resident physician/Intern/ Fellow	38 (19.6%)
	Specialist physician with less than 5 years’ experience	47 (24.2%)
	Specialist physician with more than 5 years’ experience	88 (45.4%)
	Head of Department/Professor	21 (10.8%)

**Table 2:** Epidemiological and general data regarding COVID-19.

Questions 7–12, 29–30 of the Physicians’ Perspectives on COVID-19 Questionnaire		
7	Do you work in a service/department/clinic dedicated to/or where patients with COVID-19 are hospitalized?	
	Yes	92 (47.4%)
	No	102 (52.6%)
8	Do you believe you might come in contact with COVID-19 infected patients during consultations/ medical procedures?	
	Yes	159 (82.0%)
	No	11 (5.7%)
	Not sure	24 (12.4%)
9	After starting to work in units where patients diagnosed with COVID-19 infection were hospitalized, did you feel avoided/rejected by the persons with whom you usually interact in daily life?	
	Yes	41 (21.1%)
	No	102 (52.6%)
	Not applicable	51 (26.3%)
10	Have you already had COVID-19 yourself?	
	No	120 (61.9%)
	Yes, an asymptomatic form	4 (2.1%)
	Yes, a symptomatic form	8 (4.1%)
	Not sure	62 (32%)
11	Has any of your household members had COVID-19?	
	No	136 (70.1%)
	Yes, an asymptomatic form	1 (0.5%)
	Yes, a symptomatic form	7 (3.6%)
	Not sure	50 (25.8%)
12	Do you believe that the protective equipment, and clinic’s procedures in triage and differentiated pathways, are enough to protect you?	
	Yes	71 (36.6%)
	No	63 (32.5%)
	Not sure	60 (30.9%)
29	Do you think that COVID-19 free patients are medically neglected during this period?	
	Yes	141 (72.7%)
	No	32 (16.5%)
	Not sure Missing	20 (10.3%) 1 (0.5%)
30	Are you currently isolated from the people with whom you usually live with?	
	Yes	48 (24.7%)
	No Missing	144 (74.2%) 2 (1.0%)

**Table 3:** Clinical and management selected options.

Questions 13–20, 24 of the Physicians’ Perspectives on COVID-19 Questionnaire				
13 *	Which of the following clinical symptoms do you think are suggestive for COVID-19?			
	Fever	192 (92.0%)	Anorexia	44 (22.7%)
	Cough	190 (97.9%)	Chest pain	108 (55.7%)
	Dyspnea	189 (97.4%)	Cutaneous eruptions	66 (34.0%)
	Anosmia/ ageusia	167 (86.1%)	Conjunctivitis	57 (29.4%)
	Abdominal pain	77 (39.7%)	Headache	125 (64.4%)
	Diarrhea	136 (70.1%)	Dysuria	4 (2.1%)
14 *	Which of the following tests has the highest diagnostic accuracy, in your opinion?			
	RT-PCR SARS-CoV2 of the nasopharyngeal secretion	126 (64.9%)	IgG SARS-CoV2	8 (4.1%)
	RT-PCR SARS-CoV2 of stool sample	5 (2.6%)	Chest X-ray	2 (1.0%)
	RT-PCR SARS-CoV2 of the conjunctival secretion	0 (0.0%)	Chest CT (computed tomography)	40 (20.6%)
	IgM SARS-CoV2	8 (4.1%)	Other	0 (0.0%)
15 *	Which of the following associated comorbidities are negative prognostic factors for COVID-19, in your opinion?			
	Arterial hypertension	145 (74.7%)	Liver cirrhosis	90 (46.4%)
	Heart failure	159 (82.0%)	Autoimmune pathology	97 (50.0%)
	Chronic respiratory failure	173 (89.2%)	Neoplasia	114 (58.8%)
	Chronic kidney disease (without dialysis)	91 (46.9%)	Obesity	149 (76.8%)
	Chronic kidney disease (with dialysis)	143 (73.7%)	COVID-19 disease course is not related to comorbidities	0 (0.0%)
	Diabetes mellitus	166 (85.6%)		
16 *	Which of the following blood test you think it is correlated to outcomes in COVID-19?			
	Ferritin	69 (35.6%)	NT-proBNP	39 (20.1%)
	C-reactive protein	115 (59.3%)	D-dimers	125 (64.4%)
	Lymphocyte counts	125 (64.4%)	SARS-CoV-2 viral load	82 (42.3%)
	Troponin	60 (30.9%)	None	5 (2.6%)
			Other	10 (5.2%)
17 *	Which of the following do you think are potential transmission pathways for SARS-CoV-2 virus?			
	Respiratory	192 (99.0%)	Contact with contaminated objects	168 (86.6%)
	Fecal-oral route	91 (46.9%)	Others	4 (2.0%)
18 *	Do you think there is any useful therapy for the prevention of COVID-19?			
	Vitamin D	40 (20.6%)	Astragalus extract	3 (1.5%)
	Zinc	35 (18.0%)	Quercetin	2 (1.0%)
	Vitamin C	45 (23.2%)	N-acetyl Cysteine	6 (3.1%)
	Hydroxychloroquine	33 (17.0%)	None	113 (58.2%)
			Others	
19 *	Which of the following therapies do you think is effective in COVID-19?			
	No effective treatment exists	69 (35.6%)	Azithromycin	55 (28.4%)
	Paracetamol	42 (21.6%)	Tocilizumab	43 (22.2%)
	Lopinavir/ Ritonavir	32 (16.5%)	Remdesivir	62 (32%)
	Oseltamivir	10 (5.2%)	Plasma from convalescent donors	94 (48.5%)
	Hydroxychloroquine	64 (33%)	Something else	17 (8.7%)
20 *	Is there any pre-existing medication that might worsen the prognosis of the COVID-19?			
	None	26 (13.4%)	Corticosteroids	65 (33.5%)
	NSAIDs	72 (37.1%)	Immunosuppressive drugs	119 (61.3%)
	ACE inhibitors	51 (26.3%)	Other (please specify)	6 (3.0%)
	Sartans	11 (5.7%)		
24	Do you think abnormal hemostasis plays a central role in the pathogenesis of COVID-19 and should be targeted by therapeutic anticoagulation?			
	Yes		110 (56.7%)	
	No		11 (5.7%)	
	Not sure Missing		72 (37.1%) 1 (0.5%)	

* Multiple answers.

**Table 4:** Future developments regarding the COVID-19 outcome.

Questions 21–23,25–26 of the Physicians’ Perspectives on COVID-19 Questionnaire.		
21	Do you think we will have an effective vaccine to prevent the COVID-19?	
	No	45 (23.2%)
	Yes, within the next 3 months	1 (0.5%)
	Yes, within the next 3–6 months	12 (6.2%)
	Yes, within the next 6–12 months	50 (25.8%)
	Yes, after at least 12 months Missing	85 (43.8%) 1 (0.5%)
22	Do you think we will have an effective antiviral treatment for COVID-19?	
	No	51 (26.3%)
	Yes, within the next 3 months	11 (5.7%)
	Yes, within the next 3–6 months	33 (17.0%)
	Yes, within the next 6–12 months	36 (18.6%)
	Yes, after at least 12 months Missing	62 (32.0%) 1 (0.5%)
23	Do you think that reinfections with COVID-19 can occur?	
	No	27 (13.9%)
	Yes, in less than 6 months with the same viral strain	60 (30.9%)
	Yes, after at least 6 months with the same viral strain	24 (12.4%)
	Yes, after at least 12 months with the same viral strain	9 (4.6%)
	Yes, after at least 12 months with a new mutated viral strain Missing	71 (36.6%) 3 (1.5%)
25	Do you think it will be possible to eradicate COVID-19 infection?	
	No, it will remain a permanent viral infection in the population	125 (64.4%)
	Yes, within 3 months	1 (0.5%)
	Yes, within 6 months	3 (1.5%)
	Yes, within 12 months	13 (6.7%)
	Yes, after at least 24 months	51 (26.3%)
26	Dou you think it is possible to have persistence of SARS-CoV-2 in the human body with subsequently periodic reactivation?	
	Yes	47 (24.2%)
	No	44 (22.7%)
	Not sure	101 (52.1%)

**Table 5:** Source of medical information and general data.

Questions 27–28 of the Physicians’ Perspectives on COVID-19 Questionnaire		
27	Do you consider that you receive enough information about COVID-19?	
	Yes	106 (54.6%)
	No	40 (20.6%)
	Not sure	47 (24.2%)
28	Where do you get medical/scientific information about COVID-19?	
	Medical journals	167 (86.1%)
	Scientific societies websites	160 (82.5%)
	Internal hospital protocols at workplace	127 (65.5%)
	Hospital protocols from other than workplace	84 (43.3%)
	Social media	93 (47.9%)
	Other (please specify)	9 (4.5%)

## Supplementary Materials

The following are available online at https://www.mdpi.com/2227-9032/8/3/250/s1, FILE S1: The Questionnaire of Physicians’ Perspective on COVID-19, FILE S2: The distribution based on the country and medical specialty, Table S1: Question 4—Which country do you currently practice medicine? Table S2: Question 5—What is your medical specialty? Figure S1: Question 4—Which country do you currently practice medicine? Figure S2: World Map—Question 4—Which country do you currently practice medicine, Figure S3: Question—What is your medical specialty?
